# Inhibitory Effects on the Polyol Pathway in Type 2 Diabetic Rats by Chickpea Flavonoid Extract

**DOI:** 10.3390/foods15142573

**Published:** 2026-07-22

**Authors:** Jingteng Wang, Ting Yang, Jintian Guo, Yuan Li, Yinghua Fu

**Affiliations:** 1 College of Smart Agriculture (Research Institute), Xinjiang University, Urumqi 830017, China; 18769520685@163.com (J.W.); xjuyt6122@163.com (T.Y.); guojintian99@163.com (J.G.); 2 Research Center of Food Colloids and Delivery of Functionality, College of Food Science and Nutritional Engineering, China Agricultural University, Beijing 100083, China

**Keywords:** chickpea flavonoid extract, hypoglycemic effect, polyol pathway, oxidative stress, serum metabolomics analysis

## Abstract

Chickpea is an important source of plant flavonoids in the diet, and flavonoids from chickpea have hypoglycemic activity. In this study, a male SD rat model of type 2 diabetes mellitus (T2DM) induced by a high-fat high-sugar diet combined with streptozotocin (STZ) was used to investigate the inhibitory effects on the polyol pathway (a branch of glucose metabolism) by chickpea flavonoid extract (CFE). The results demonstrated that CFE significantly lowered fasting blood glucose (FBG) level, and reduced insulin resistance in diabetic rats by elevating the homeostasis model assessment of insulin sensitivity (HOMA-IS) and decreasing the homeostasis model assessment of insulin resistance (HOMA-IR). And CFE relieved oxidative stress through reducing H_2_O_2,_ malondialdehyde (MDA) and carbonylated protein levels, and increasing the activity of glutathione peroxidase (GSH-Px). Moreover, CFE inhibited the polyol pathway by downregulating the aldose reductase (AR) and sorbitol dehydrogenase (SDH) activities, as well as reducing the sorbitol and fructose levels. Meanwhile, CFE also enhanced the antioxidant defense capacity through increasing glutathione reductase (GR) activity and the glutathione (GSH) level, while decreasing the oxidized glutathione (GSSG) level. Further results showed that CFE mitigated reductive stress in T2DM rats via increasing intracellular NAD^+^ content and the NAD^+^/NADH ratio, due to suppressing PARP activity and upregulating Sirt3 activity. Furthermore, CFE regulated the levels of metabolites such as nicotinamide and *β*-aminobutyric acid, and modulated seven metabolic pathways closely associated with the improvement of diabetes and its complications. Ten key differential metabolites were reversed after CFE intervention, which were strongly correlated with the polyol pathway and oxidative stress in T2DM rats. In summary, CFE possessed hypoglycemic activity and could inhibit the polyol pathway, which was considered a promising natural product for diabetes prevention.

## 1. Introduction

Diabetes and its complications are diseases caused by impaired glucose metabolism [1], and the condition is diagnosed by detecting elevated blood glucose levels [2,3]. Currently, diabetes is primarily treated with medication, but drug therapy is associated with certain side effects [4]. Therefore, the extraction of hypoglycemic active compounds from natural products has garnered increasing attention. Under hyperglycemic conditions, multiple branches of glucose metabolic pathways which typically remain dormant are activated, and these pathways include the polyol pathway, the aminohexose pathway, the protein kinase C (PKC) activation pathway, the advanced glycation end-product (AGE) pathway, and the enediol pathway [5]. Under hyperglycemic conditions, at least 30% of glucose is metabolized via the polyol pathway: firstly glucose is reduced to sorbitol by aldose reductase (AR) with coenzyme NADPH and produces NADP^+^, then sorbitol is oxidized to fructose by dehydrogenase (SDH) with coenzyme NAD^+^ and produces NADH [6,7]. Under hyperglycemic conditions, activation of the polyol pathway consumes NAD^+^, leading to a decrease in the NAD^+^ level in the cytosol, and this is considered a key step in causing NAD^+^/NADH redox imbalance and cellular hypoxia, impairing NADH oxidation or NAD^+^ regeneration [8]. Moreover, in the antioxidant defense system, oxidized glutathione (GSSG) can be converted into reduced glutathione (GSH) under glutathione reductase (GR) and coenzyme NADPH. However, the consumption of NADPH due to the activation of the polyol pathway under hyperglycemic conditions leads to reducing glutathione reductase activity, consequently diminishing antioxidant defense capacity [6,7]. Furthermore, intermediates or end products generated by the polyol pathway can exacerbate diabetes and its complications. Sorbitol can accumulate in retinal and renal tissues, leading to osmotic stress and cell death [9,10], while fructose can cause non-enzymatic glycation or nitrosation of proteins [11] and contribute to the development of non-alcoholic fatty liver disease [12]. Kato et al. found that ginger extract can inhibit aldose reductase activity in diabetic rats, helping to prevent or improve diabetes and its complications [13]. Fu et al. demonstrated that the degree of NAD^+^/NADH redox imbalance in the pancreas of T2DM rats was alleviated by crude chickpea flavonoid extract (CCFE), which was likely attributed to the inhibition of the polyol pathway and the decrease in poly ADP ribose polymerase (PARP) and sirtuin 3 (Sirt3) activities [14].

Numerous studies have reported that legumes and chickpeas are rich in flavonoids, which possess definite hypoglycemic properties. Yang et al. established an insulin resistance (IR) rat model, and found that oxytropis total flavonoids reduced fasting blood glucose (FBG) and 2 h postprandial blood glucose (PBG) levels, and stimulated insulin secretion in IR rats [15]. Li et al. synthesized four novel isoflavone derivatives from chickpeas, and found that the target compounds genistein, biochanin A, formononetin and genistein/chromium chelate complex of isoflavone exhibited improvement of insulin-resistant activity in an insulin-resistant HepG2 cell model [16]. Gao et al. found that chickpea isoflavones inhibited the differentiation and lipid accumulation of 3T3-L1 preadipocytes, and stimulated glucose uptake by downregulating the mRNA expression of peroxisome proliferator-activated receptor γ(PPARγ), CCAAT/enhancer-binding protein α (C/EBPα), adipocyte fatty acid-binding protein (aP2) [17], etc. Zhou et al. found that a total of twenty-nine chickpea flavonoids were identified by UPLC-MS/MS, and chickpea flavonoids reduced oxidative stress through the activation of insulin receptor substrate 1 (IRS1), phosphoinositide 3-kinase (PI3K), and phosphorylated protein kinase B (Akt) in HepG2 cells [18].

Mulei County in Xinjiang is a major production area for chickpeas in China, accounting for more than 83% of total chickpea cultivation area [19]. Chickpeas are highly nutritious, rich in flavonoids, and possess hypoglycemic activity [20]. Aldose reductase (AR), the rate-limiting enzyme in the polyol pathway, may be a causative factor in diabetes and its complications. In this study, a T2DM rat model developed through a high-fat, high-sugar diet combined with streptozotocin (STZ) was established to investigate the hypoglycemic effects and alleviating oxidative stress in T2DM rats by chickpea flavonoid extract (CFE). Simultaneously, the influence of the chickpea flavonoid extract on the polyol pathway and redox stress in T2DM rats was investigated. Furthermore, metabolomic analysis was performed to explore the regulating metabolites in T2DM rats by CFE.

## 2. Materials and Methods

### 2.1. Plant Materials, Chemicals and Reagents

The kabuli chickpeas were purchased from Yingge Biotechnology Co., Ltd. (Mulei County, China) in 2024. After removing damaged and diseased grains, the chickpeas were pulverized, passed through an 80-mesh sieve and dried at 50 °C for 3 h, then stored at 4 °C for later use. The STZ used in the present study was purchased from (Sigma-Aldrich, St. Louis, MO, USA), and rutin (HPLC ≥ 98%) was purchased from Solarbio (Beijing, China). All the other chemicals used in the present work were at least of analytical grade.

### 2.2. Extraction of CFE

The chickpea flavonoid extract (CFE) was extracted and enriched following previous laboratory methods [21]. Briefly, a total of 150.00 g of dried chickpea powder was defatted with petroleum ether at a solid/liquid ratio of 1:10 for 12 h to obtain defatted chickpea powder. Subsequently, 70% ethanol solution was added at a solid/liquid ratio of 1:20, and the mixture was subjected to reflux extraction in a water bath at 80 °C for 4 h. After suction filtration, the filtrate was collected as the crude chickpea total flavonoid extract. AB-8 macroporous resin was used for further enrichment under the following conditions: loading concentration of 0.4 mg/mL and elution with 70% ethanol. Based on the rutin standard curve, the total flavonoid content (TFC) in the chickpea flavonoid extract (CFE) was 52.5648 ± 0.3870 mg/g.

### 2.3. Animals

The experimental protocols were approved by the Animal Ethics Committee of the Newlong Youshu Life Sciences Co., Ltd. (Hangzhou, China, approval number: YS-m202511010). Male SD rats (160 ± 15 g) were purchased from the Newlong Youshu Life Sciences Co., Ltd. (Hangzhou, China). All rats were kept under controlled temperatures (25 ± 2 °C) and relative humidity (50–70%) on a 12/12 h light/dark cycle with free access to water and a normal diet for 7 days before the experiment.

### 2.4. Establishment of the T2DM Model in Rats

After one week of adaptive feeding, 10 rats were randomly selected as the normal control group (NC), which were fed with basal diet, and the remaining 50 rats received a high-fat and high-sugar diet (HFHS) for four weeks. After fasting for 12 h, all HFHS-fed rats were intraperitoneally injected with a single dose of streptozotocin (STZ, 45 mg/kg), and the NC group were injected with an equal volume of 0.1 mol/L sodium citrate/citric acid buffer solution. The NC group were continuously fed with a basal diet, and the diabetic model groups were fed with a high-fat and high-sugar diet. One week later, all rats were fasted for 12 h without water deprivation. Blood samples were collected from the tail vein to measure fasting blood glucose (FBG). Rats with FBG levels exceeding 11.1 mmol/L were considered successfully established T2DM models.

### 2.5. Treatment of T2DM Rats with CFE

**Figure 1:**
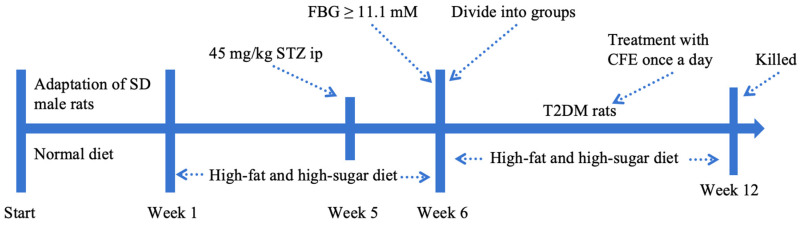
Animal experimental design.

The qualified diabetic rats were randomly divided into five groups, each group consisting of 10 rats: diabetic model group (DM), positive control group (PC) and three CFE-treated groups. Metformin powder was dissolved in normal saline for intragastric administration, and rats in the PC group were gavaged at a daily dose of 200 mg/kg·BW metformin. The corresponding daily gavage doses for the low-dose chickpea flavonoid extract group (CFE-L), medium-dose CFE group (CFE-M) and high-dose CFE group (CFE-H) were 130, 390 and 650 mg/kg·BW, respectively. Rats in the NC and DM groups were administered an equal volume of normal saline. All interventions were performed once daily for six weeks. The experimental procedure is shown in Figure 1.

### 2.6. Sample Collection and Hematoxylin and Eosin (HE) Staining

After euthanasia, the pancreatic tissues of rats were rapidly isolated and placed in ice-cold normal saline. The samples were fixed in 10% formaldehyde solution at room temperature for one week, followed by paraffin embedding. Subsequently, hematoxylin/eosin (HE) staining was performed. After section labeling, the pathological changes of pancreatic tissues were observed under an optical microscope at a magnification of ×400.

### 2.7. Determination of Biochemical Parameters

The fasting blood glucose (FBG) of the rats was detected using a blood glucose meter (Hengsheng Medical Technology Co., Ltd., Urumqi, China). The level of fasting insulin (FINS) in serum was measured with an ELISA kit purchased from Beijing Box Bioengineering Co., Ltd. (Beijing, China), and the indicators were calculated with the following formulas: HOMA-IS = ln [1/(FINS × FBG)], HOMA-IR = (FINS × FBG)/22.5. The contents of total cholesterol (TC), triglyceride (TG), low-density lipoprotein cholesterol (LDLC), high-density lipoprotein cholesterol (HDLC), hydrogen peroxide (H_2_O_2_), malondialdehyde (MDA) and the activity of glutathione peroxidase (GSH-Px) in serum were determined using kits purchased from Nanjing Jiancheng Bioengineering Institute (Nanjing, China). The content of protein carbonylation (PCO) in pancreatic and liver tissues was detected with a kit purchased from Beijing Box Bioengineering Co., Ltd. The content of fructose as well as the activities of alanine aminotransferase (ALT) and aspartate aminotransferase (AST) in serum were measured using kits purchased from Beijing Box Bioengineering Co., Ltd. The activities of aldose reductase (AR), succinate dehydrogenase (SDH), glutathione reductase (GR), poly ADP-ribose polymerase (PARP) and sirtuin3 (Sirt3), as well as the contents of sorbitol, coenzyme NAD(H), oxidized glutathione (GSSG) and reduced glutathione (GSH) in pancreatic and liver tissues were detected using kits purchased from Beijing Box Bioengineering Co., Ltd. All measurements were conducted in strict accordance with the manufacturer’s instructions using assay kits.

### 2.8. Metabolomics Analysis

LC-MS/MS technology was applied to analyze differential metabolites in the serum of T2DM rats. Serum samples were thawed at 4 °C and vortexed for 1 min. Next, 2 mL of each serum sample was transferred and mixed with 400 μL of methanol. The mixture was centrifuged at 12,000 rpm for 10 min, and the resulting supernatant was collected. Then 150 μL of 2-chloro-L-phenylalanine was added to the supernatant for dissolution. Finally, the solution was filtered through a 0.22 μm filter membrane and the filtrate was collected for LC-MS/MS analysis. The metabolomics data were entered into RXCMS 4.9.1 after initial data analysis using Proteowizard 3.0 software.

### 2.9. Statistical Analysis

The data results of all the experiments were represented as mean ± SD. Variances among groups were analyzed by one-way analysis of variance (ANOVA) using IBM SPSS Statistics 26, with *p* < 0.05 regarded as statistically different, and graphing was performed using GraphPad Prism 9.5.

## 3. Results and Discussion

### 3.1. Effects of CFE on Fasting Blood Glucose (FBG) and Insulin Resistance in T2DM Rats

**Figure 2:**
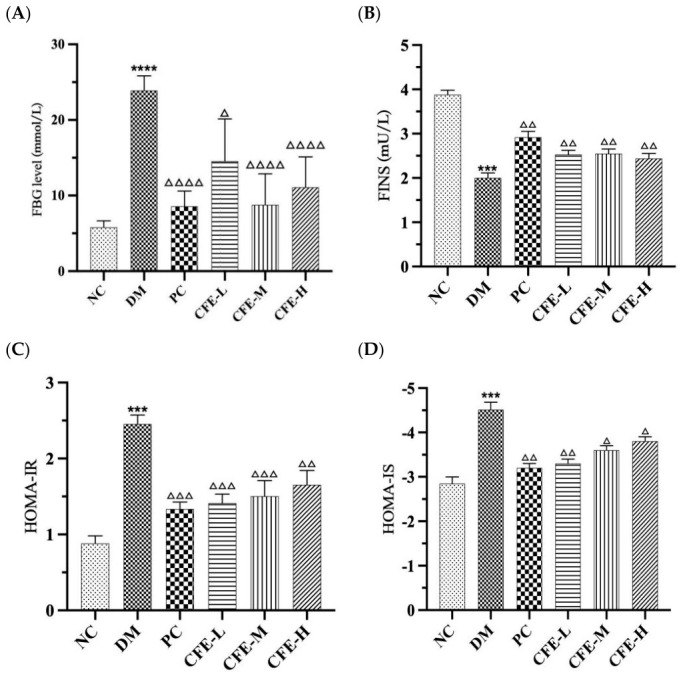
Effects of CFE on FBG, FINS, HOMA-IR, and HOMA-IS levels in T2DM rats. (**A**) FBG; (**B**) FINS; (**C**) HOMA-IR; (**D**) HOMA-IS. *** *p* < 0.001 and **** *p* < 0.0001, compared with the NC group; Δ *p* < 0.05, ΔΔ *p* < 0.01, ΔΔΔ *p* < 0.001 and ΔΔΔΔ *p* < 0.0001, compared with the DM control group. NC: normal control group, DM: diabetic model control group, PC: positive control group, CFE-(L, M, H): (low, middle, high) dose CFE-treated group.

In diabetes, insulin resistance may occur, so the hypoglycemic effect of CFE and its improvement on insulin resistance in T2DM rats was investigated, and the results are shown in Figure 2. After 6 weeks of intervention, compared with the diabetic model group (DM), the fasting blood glucose levels in the CFE-L, CFE-M and CFE-H groups were significantly decreased (*p* < 0.05) (Figure 2A). As shown in Figure 2B–D, serum fasting insulin (FINS) levels were markedly increased (*p* < 0.01), the HOMA-IR index was significantly decreased (*p* < 0.01), and the HOMA-IS index was effectively elevated (*p* < 0.05) in the CFE-L, CFE-M and CFE-H groups. Rutin could significantly reduce fasting blood glucose levels in STZ-induced diabetic rats by inhibiting key enzymes involved in hepatic gluconeogenesis and enhancing insulin sensitivity in peripheral tissues [22]. Quercetin markedly decreased fasting blood glucose in STZ-induced diabetic rats via activating the pancreatic Nrf2 antioxidant pathway and alleviating oxidative damage of pancreatic β-cells [23].

### 3.2. Effects of CFE on Pancreatic HE Staining in T2DM Rats

**Figure 3:**
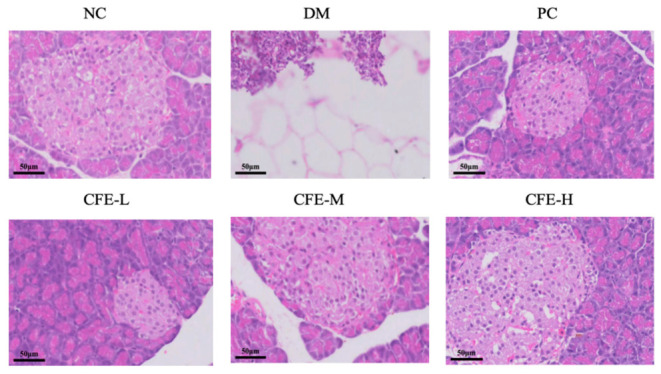
Histological sections of the pancreas of the normal control and T2DM rats (hematoxylin and eosin histochemical staining). Scale bar: 50 µm. NC: normal control group, DM: diabetic model control group, PC: positive control group, CFE-(L, M, H): (low, middle, high) dose CFE-treated group.

Hematoxylin/eosin (HE) staining was used to evaluate the morphological changes of pancreatic tissues in T2DM rats. As shown in Figure 3, the pancreatic tissue of rats in the NC presented intact morphology with clear islet structure, closely arranged and abundant islet cells, and plump cytoplasm. In the DM, pancreatic islets were severely damaged due to the high-fat and high-sugar diet combined with STZ administration, manifesting as obviously shrunken and irregular islets, reduced number and loosely arranged islet cells, along with vacuolar degeneration and apoptotic characteristics. After 6 weeks of CFE intervention, the injuries of islets and pancreatic β-cells in the CFE-L, CFE-M and CFE-H groups were alleviated.

### 3.3. Effects of CFE on Oxidative Stress in T2DM Rats

**Figure 4:**
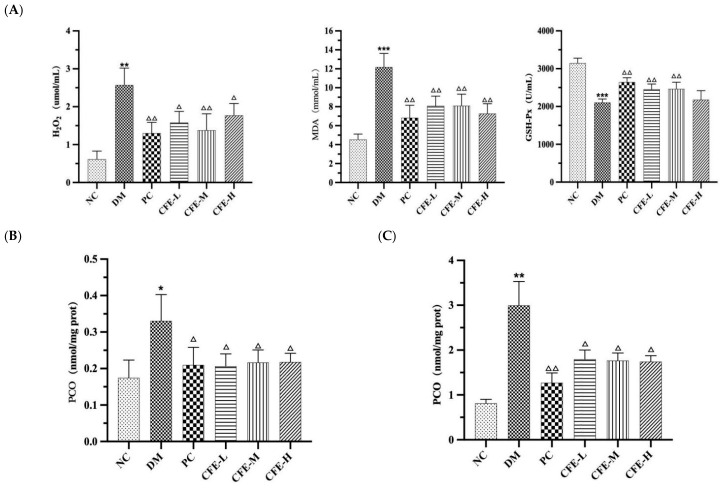
Effects of CFE on oxidative stress in T2DM rats. (**A**) H_2_O_2_; MDA; GSH-Px; (**B**) pancreatic tissue PCO; (**C**) hepatic tissue PCO. * *p* < 0.05, ** *p* < 0.01 and *** *p* < 0.001, compared with the NC group; Δ *p* < 0.05 and ΔΔ *p* < 0.01, compared with the DM control group. NC: normal control group, DM: diabetic model control group, PC: positive control group, CFE-(L, M, H): (low, middle, high) dose CFE-treated group.

As shown in Figure 4A, compared with the diabetic model group (DM), the serum H_2_O_2_ and MDA levels were significantly decreased (*p* < 0.05) in the CFE-L, CFE-M and CFE-H groups, and the serum GSH-Px activities were significantly increased in the CFE-L and CFE-M groups (*p* < 0.01). According to Figure 4B,C, the carbonylated protein levels in pancreatic and hepatic tissues were notably downregulated in all CFE intervention groups (*p* < 0.01). Puerarin could activate the Nrf2/HO-1 signaling pathway in the retinal tissue of diabetic rats, reduce MDA levels and enhance GSH-Px activity, thereby alleviating oxidative stress damage [24]. Quercetin ameliorated oxidative stress in the liver and nerve tissues of galactose-induced hyperglycemic rats, decreased the level of protein carbonylation, and protected tissues against oxidative injury [25].

### 3.4. Effects of CFE on the Polyol Pathway in T2DM Rats

**Figure 5:**
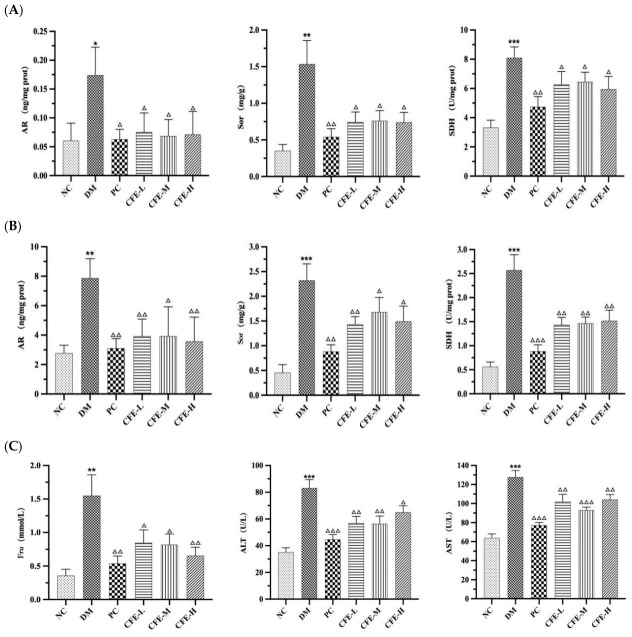
Effects of CFE on polyol pathway in T2DM rats. (**A**) Pancreatic tissue AR, Sor, SDH; (**B**) hepatic tissue AR, Sor, SDH; (**C**) serum Fru, ALT, AST. * *p* < 0.05, ** *p* < 0.01 and *** *p* < 0.001, compared with the NC group; Δ *p* < 0.05, ΔΔ *p* < 0.01 and ΔΔΔ *p* < 0.001, compared with the DM control group. NC: normal control group, DM: diabetic model control group, PC: positive control group, CFE-(L, M, H): (low, middle, high) dose CFE-treated group.

Aberrant activation of the polyol pathway is one of the crucial pathological mechanisms underlying T2DM and its complications. This pathway mainly relies on the catalytic effects of aldose reductase (AR) and sorbitol dehydrogenase (SDH). It remains relatively quiescent under physiological conditions, yet becomes highly activated as an alternative glucose metabolic pathway in the setting of hyperglycemia [26]. The inhibitory effect of chickpea flavonoid extract (CFE) on the polyol pathway in T2DM rats is shown in Figure 5A–C. Compared with the NC, the activity of AR in pancreatic and hepatic tissues was significantly increased in the DM (*p* < 0.05), indicating that the normally dormant polyol pathway was activated in T2DM rats induced by high-fat and high-sugar diet combined with STZ injection. After supplementation with CFE, compared with the DM, the activities of aldose reductase (AR) and sorbitol dehydrogenase (SDH) in the pancreatic and liver tissues of T2DM rats in the CFE-L, CFE-M and CFE-H groups were significantly decreased (*p* < 0.05). Meanwhile, the levels of sorbitol and fructose in serum were markedly reduced (*p* < 0.01), and alanine transaminase (ALT) and aspartate transaminase (AST) levels in serum were also significantly declined (*p* < 0.05). Quercetin could potently inhibit the activity of aldose reductase (AR) in rat lenses, suppress the polyol pathway, and alleviate hyperglycemia-induced tissue damage and diabetic complications [27]. Quercetin inhibited aldose reductase (AR) activity and reduced sorbitol production in rat lenses, thereby relieving oxidative stress and diabetic complications under hyperglycemic conditions [28]. In summary, CFE could inhibit the polyol pathway, a critical bypass of glucose metabolism, and protect liver tissues in T2DM rats.

### 3.5. Effects of CFE on Glutathione Reductase in T2DM Rats

**Figure 6:**
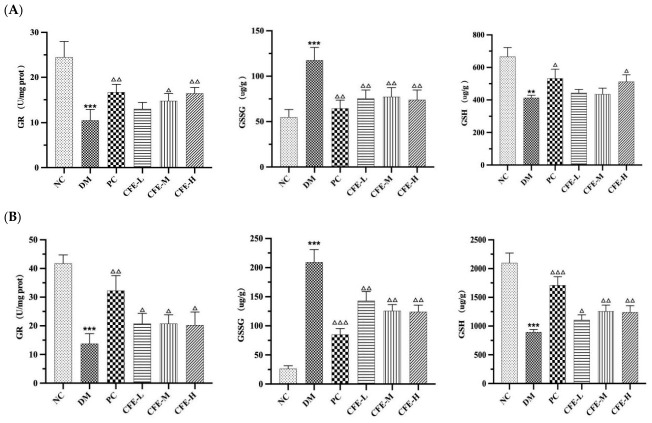
Effects of CFE on glutathione reductase in T2DM rats. (**A**) Pancreatic tissue GR, GSSG, GSH; (**B**) hepatic tissue GR, GSSG, GSH. ** *p* < 0.01, *** *p* < 0.001, compared with the NC group; Δ *p* < 0.05, ΔΔ *p* < 0.01 and ΔΔΔ *p* < 0.001, compared with the DM control group. NC: normal control group, DM: diabetic model control group, PC: positive control group, CFE-(L, M, H): (low, middle, high) dose CFE-treated group.

Activation of the polyol pathway consumes excessive NADPH, inhibits glutathione reductase activity, and consequently weakens the antioxidant defense capacity of the body. As shown in Figure 6A,B, compared with the DM, CFE treatment notably upregulated glutathione reductase (GR) activity (*p* < 0.05), downregulated oxidized glutathione (GSSG) content (*p* < 0.01), and elevated reduced glutathione (GSH) levels (*p* < 0.05). Quercetin significantly increased the level of reduced glutathione (GSH) and enhanced glutathione reductase (GR) activity in rat liver tissues, maintained GSH/GSSG homeostasis, and thus alleviated ultraviolet A-induced hepatic oxidative stress in rats [29]. Puerarin elevated hepatic glutathione reductase (GR) activity and GSH content, reduced the GSSG level, and regulated glutathione redox balance in lead-exposed rats, thereby protecting rat tissues against oxidative damage [30]. Collectively, the above results indicated that chickpea flavonoid extract (CFE) markedly upregulated glutathione reductase (GR) activity, decreased the oxidized glutathione (GSSG) level, and increased the reduced glutathione (GSH) level in pancreatic and hepatic tissues of T2DM rats, ultimately enhancing the antioxidant defense capacity.

### 3.6. Effects of CFE on Reductive Stress in T2DM Rats

**Figure 7:**
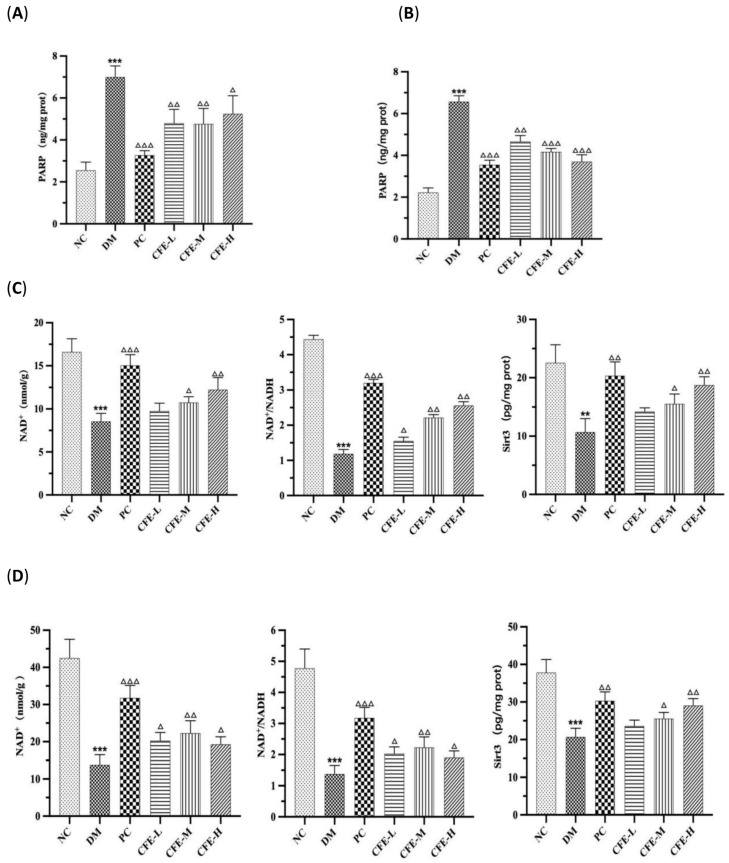
Effects of CFE on reductive stress in T2DM rats. (**A**) Pancreatic tissue PARP; (**B**) hepatic tissue PARP; (**C**) pancreatic tissue NAD^+^, NAD^+^/NADH, Sirt3; (**D**) hepatic tissue NAD^+^, NAD^+^/NADH, Sirt3. ** *p* < 0.01, *** *p* < 0.001, compared with the NC group; Δ *p* < 0.05, ΔΔ *p* < 0.01 and ΔΔΔ *p* < 0.001, compared with the DM control group. NC: normal control group, DM: diabetic model control group, PC: positive control group, CFE-(L, M, H): (low, middle, high) dose CFE-treated group.

Activation of the polyol pathway under diabetic conditions consumes NAD^+^ during the conversion of sorbitol to fructose catalyzed by sorbitol dehydrogenase. Meanwhile, NAD^+^ consumption is further intensified during the repair of damaged DNA by PARP and the deacetylation of macromolecular proteins by Sirt3. These three pathways are considered the major contributors to the imbalance of the NAD^+^/NADH redox state [31]. PARP-1 is a major enzyme responsible for DNA repair. As shown in Figure 7A–D, compared with the DM, the NAD^+^ content and NAD^+^/NADH ratio were increased significantly (*p* < 0.05), PARP activity was reduced (*p* < 0.05), and Sirt3 activity was markedly upregulated (*p* < 0.05) in the pancreatic and hepatic tissues in T2DM rats in the CFE-L, CFE-M and CFE-H groups. Rutin could elevate the hepatic NAD^+^ level, reduce the NADH level, and regulate the NAD^+^/NADH redox balance in streptozotocin (STZ)-induced diabetic rats, thereby alleviating reductive stress and oxidative damage under hyperglycemic conditions [32]. These findings indicated that CFE could suppress the overactivated PARP and enhance Sirt3 activity, to elevate NAD^+^ levels and the NAD^+^/NADH ratio, ultimately alleviating reductive stress and redox imbalance in T2DM rats.

### 3.7. Multivariate Statistical Analyses and Identification of Metabolites

Subsequently, metabolomic analysis was employed to further explore the ameliorative effects of CFE on diabetes and its complications in rats. The results of principal component analysis (PCA) and orthogonal partial least squares discriminant analysis (OPLS-DA) demonstrated that the model exhibited good stability and reliability (Supporting Information: Figures S1 and S2; the fitting parameters R^2^X were 0.316 and 0.388 in positive ion mode respectively, R^2^Y were 0.999 and 0.998 in negative ion mode respectively, and prediction parameters Q^2^ were 0.760 and 0.827 respectively). According to the screening criteria of the OPLS-DA model, metabolites with variable importance in projection (VIP) values > 1 and *p* values < 0.05 were identified as statistically significant differences (Supporting Information: Tables S1 and S2). A total of 708 differential metabolites were identified between the control group and the diabetic model group, and 386 differential metabolites were identified between the model group and the CFE intervention group.

**Figure 8:**
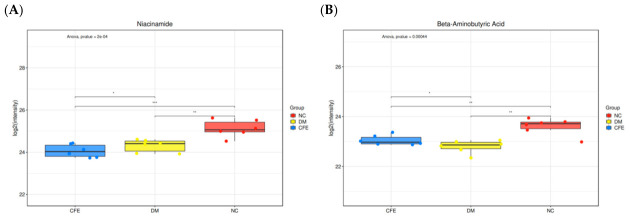
Box plots of the effects of CFE on serum differential metabolites in T2DM rats ((**A**) Niacinamide; (**B**) Beta-Aminobutyric Acid). In (**A**): * *p* < 0.05, CFE group compared with the DM control group; ** *p* < 0.01, DM control group compared with the NC group; *** *p* < 0.001, CFE group compared with the NC group. In (**B**): * *p* < 0.05, CFE group compared with the DM control group; ** *p* < 0.01, DM control group compared with the NC group; ** *p* < 0.001, CFE group compared with the NC group.

In addition, nicotinamide is a key product of the NAD^+^ metabolic pathway, and its metabolic disorder is directly associated with insulin resistance and β-cell dysfunction in T2DM. Relevant studies have confirmed that abnormal levels of nicotinamide metabolites can serve as characteristic indicators related to insulin resistance in T2DM rats [33]. And *β*-aminobutyric acid can ameliorate insulin resistance by reducing lipid accumulation and inhibiting inflammatory responses, and changes in serum level can reflect the improvement of lipid metabolism in T2DM patients [34]. Compared with the DM group, the CFE group presented a significant downregulation of nicotinamide (*p* < 0.05) (Figure 8A) and a marked upregulation of β-aminobutyric acid (*p* < 0.05) (Figure 8B). Collectively, nicotinamide and *β*-aminobutyric acid were regarded as potential biomarkers to alleviate insulin resistance and lipid metabolism disorder in T2DM rats treated with CFE.

In addition, based on the criteria of *p* < 0.05 and impact value > 0.1, KEGG enrichment analysis identified nine major metabolic pathways regulated in T2DM rats by CFE (Supporting Information: Figure S3). Among them, seven pathways were closely associated with the improvement of diabetes and its complications, including central carbon metabolism in cancer, glycolysis, phenylalanine, tyrosine and tryptophan biosynthesis, synaptic vesicle cycle, GABAergic synapse, mineral absorption, and the FoxO signaling pathway.

### 3.8. Correlation Analysis

**Figure 9:**
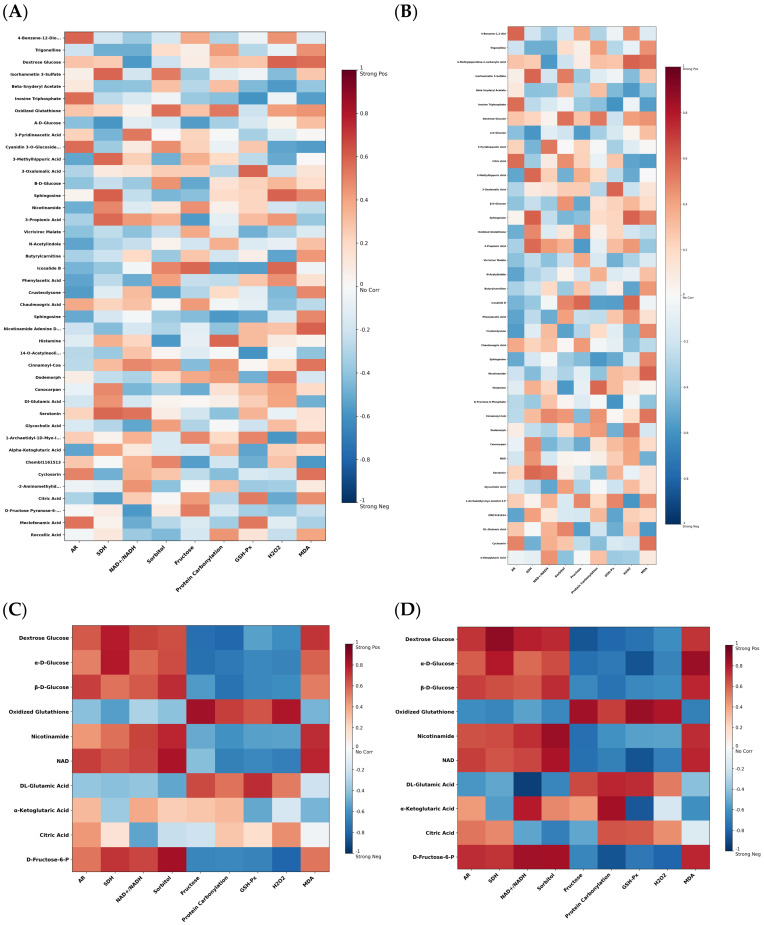
Correlation analysis of differential metabolites with polyol pathway and oxidative stress ((**A**) groups NC and DM, (**B**) groups CFE and DM); correlation analysis of key differential metabolites with polyol pathway and oxidative stress ((**C**) groups NC and DM, (**D**) groups CFE and DM).

Correlation analysis was performed between differential metabolites and indicators of the polyol pathway as well as oxidative stress (Figure 9). In the normal control group (NC) and model control group (DM), 38 differential metabolites were correlated with the polyol pathway and oxidative stress (Figure 9A). As shown in Figure 9C, key differential metabolites including D-glucose, α-D-glucose, β-D-glucose, D-fructopyranose 6-phosphate, nicotinamide and nicotinamide adenine dinucleotide were positively correlated with aldose reductase (AR) activity, sorbitol dehydrogenase (SDH) activity, sorbitol content and the NAD^+^/NADH ratio, but negatively correlated with fructose content, carbonylated protein level, GSH-Px activity, H_2_O_2_ content and MDA content. By contrast, key differential metabolites such as oxidized glutathione, DL-glutamic acid, α-ketoglutaric acid and citric acid showed positive correlations with fructose content, carbonylated protein level, GSH-Px activity, H_2_O_2_ content and MDA content, and negative correlations with AR activity, SDH activity, sorbitol content and the NAD^+^/NADH ratio.

After CFE intervention, 42 differential metabolites in the CFE and DM groups were correlated with the polyol pathway and oxidative stress (Figure 9B). As presented in Figure 9D, oxidized glutathione, DL-glutamic acid, α-ketoglutaric acid and citric acid were positively associated with fructose content, carbonylated protein level, GSH-Px activity, H_2_O_2_ content and MDA content, and negatively associated with AR activity, SDH activity, sorbitol content and the NAD^+^/NADH ratio. Meanwhile, D-glucose, α-D-glucose, β-D-glucose, D-fructopyranose-6-phosphate, nicotinamide and nicotinamide adenine dinucleotide were positively correlated with AR activity, SDH activity, sorbitol content and the NAD^+^/NADH ratio, and negatively correlated with fructose content, carbonylated protein level, GSH-Px activity, H_2_O_2_ content and MDA content.

Collectively, ten key regulated differential metabolites, namely, D-glucose, α-D-glucose, β-D-glucose, oxidized glutathione, nicotinamide, nicotinamide adenine dinucleotide, DL-glutamic acid, α-ketoglutaric acid, citric acid and D-fructopyranose 6-phosphate, were closely correlated with the polyol pathway and oxidative stress in T2DM rats. These metabolites might serve as potential biomarkers for CFE intervention in T2DM rats.

## 4. Conclusions

In conclusion, CFE could significantly reduce FBG levels, improve the insulin resist, and alleviate pancreatic tissue damage in diabetic rats. And CFE alleviated oxidative stress through reducing the contents of H_2_O_2_ and MDA, lowering carbonylated protein levels, and enhancing GSH-Px activity. Additionally, CFE inhibited the polyol pathway, a branch of glucose metabolism, by downregulating the activities of AR and SDH as well as sorbitol accumulation, while decreasing levels of fructose, ALT and AST. Moreover, CFE strengthened the antioxidant defense system by increasing GR activity and GSH content, and reducing GSSG levels. Furthermore, CFE mitigated reductive stress in T2DM rats via upregulating NAD^+^ content and the NAD^+^/NADH ratio, due to inhibiting excessive PARP activity, and elevating Sirt3 activity. Metabolomic analysis indicated that CFE modulated nicotinamide and *β*-aminobutyric acid, and markedly reversed seven metabolic pathways closely involved in improvement of diabetes and its complications, and ten key metabolites were markedly reversed following CFE intervention. In summary, chickpea flavonoid extract (CFE) might serve as a potential natural product for the prevention of diabetes and its complications.

## Data Availability

The original contributions presented in this study are included in the article. Further inquiries can be directed to the corresponding author.

## Supplementary Materials

The following supporting information can be downloaded at https://www.mdpi.com/article/10.3390/foods15142573/s1: Figure S1: Principal component analysis; Figure S2: Orthogonal projections to latent structure discriminant analysis; Figure S3: KEGG enrichment analysis of differential metabolites; Table S1: Final identified differential metabolites: NC vs. DM; Table S2: Final identified differential metabolites: CFE vs. DM.

## Abbreviations

T2DM	Type 2 diabetes mellitus
CFE	Chickpea flavonoid extract
GR	Glutathione reductase
FBG	Fasting blood glucose
AR	Aldose reductase
NAD^+^	Oxidized nicotinamide adenine dinucleotide
GSSG	Oxidized glutathione
SDH	Sorbitol dehydrogenase
NADH	Reduced nicotinamide adenine dinucleotide
NC	Normal control group
DM	Diabetic model control group
PC	Positive control group
CFE-H	High-dose CFE-treated group
CFE-M	Middle-dose CFE-treated group
CFE-L	Low-dose CFE-treated group
GSH	Reduced glutathione
H_2_O_2_	Hydrogen peroxide
MDA	Malondialdehyde
AST	Aspartate aminotransferase
GSH-Px	Glutathione peroxidase
ALT	Alanine aminotransferase
STZ	Streptozocin
PARP	Poly ADP-ribose polymerase
CCFE	Crude chickpea flavonoid extract
IRS1	Insulin receptor substrate 1
PI3K	Phosphoinositide 3-kinase
Akt	Phosphorylated protein kinase B
