# Supplementary material for: Inhibitory Effects on the Polyol Pathway in Type 2 Diabetic Rats by Chickpea Flavonoid Extract
Source: Foods. 2026 Jul 22;15(14):2573. doi: 10.3390/foods15142573 (PMC13409627; doi:10.3390/foods15142573)
Supplement: Supplementary file 1 [file foods-15-02573-s001.zip › Figure S2.pdf]

（2）Orthogonal Projections to Latent Structure Discriminant Analysis

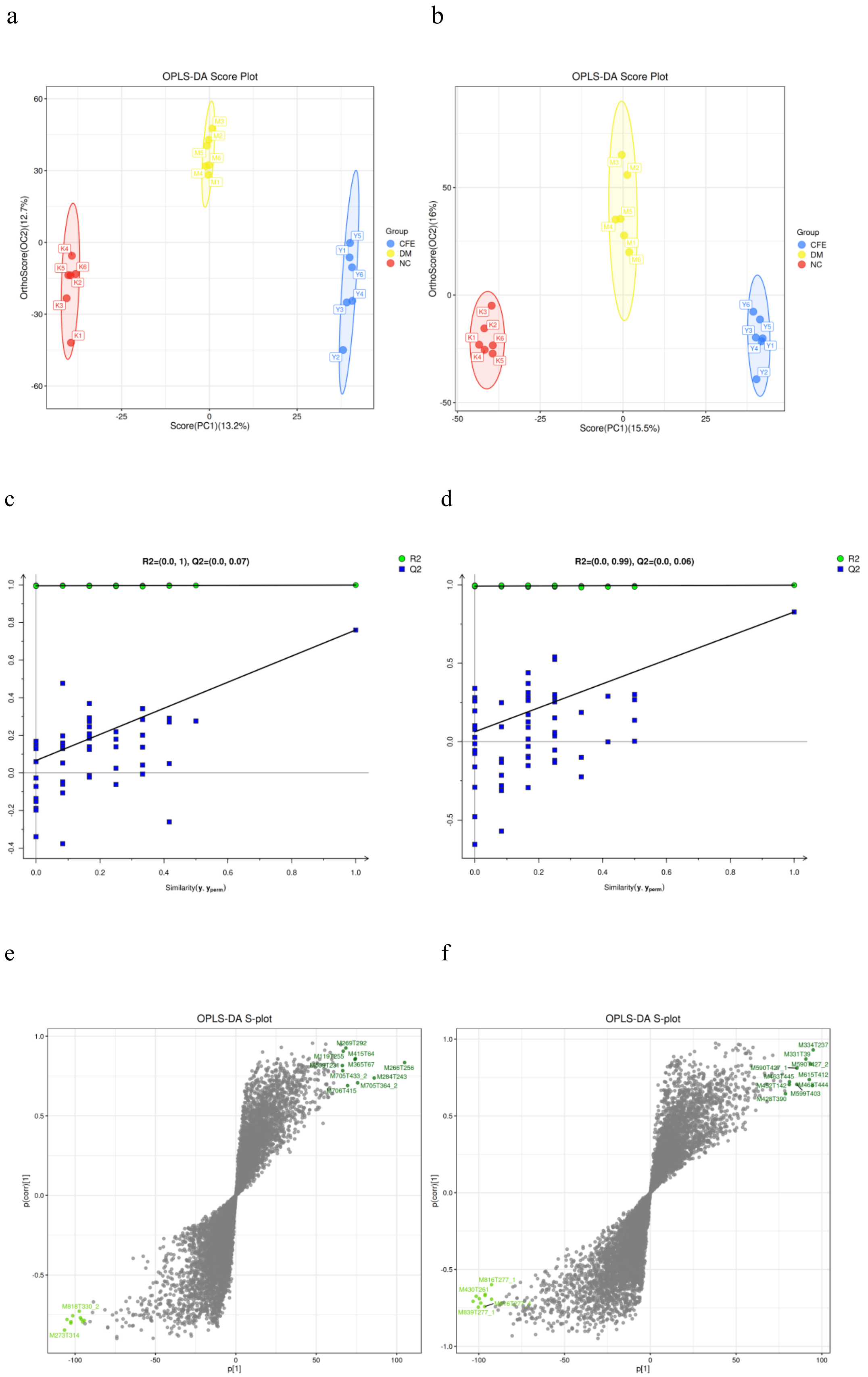

Figure.S2 OPLS-DA scores of NC, DM and CFE samples (a: positive ion mode; b: negative ion mode); OPLS-DA replacement test diagram of NC, DM and CFE samples (c: positive ion mode; d: negative ion mode); OPLS-DA S-plot for NC, DM and CFE samples (e: positive ion mode; f: negative ion mode)

Note: Red: normal control group (NC); Yellow: model control group (DM); Blue: chickpea total flavonoid extract group (CFE).
