# Supplementary material for: Inhibitory Effects on the Polyol Pathway in Type 2 Diabetic Rats by Chickpea Flavonoid Extract
Source: Foods. 2026 Jul 22;15(14):2573. doi: 10.3390/foods15142573 (PMC13409627; doi:10.3390/foods15142573)
Supplement: Supplementary file 1 [file foods-15-02573-s001.zip › Figure S3.pdf]

(3) KEGG enrichment analysis of differential metabolites

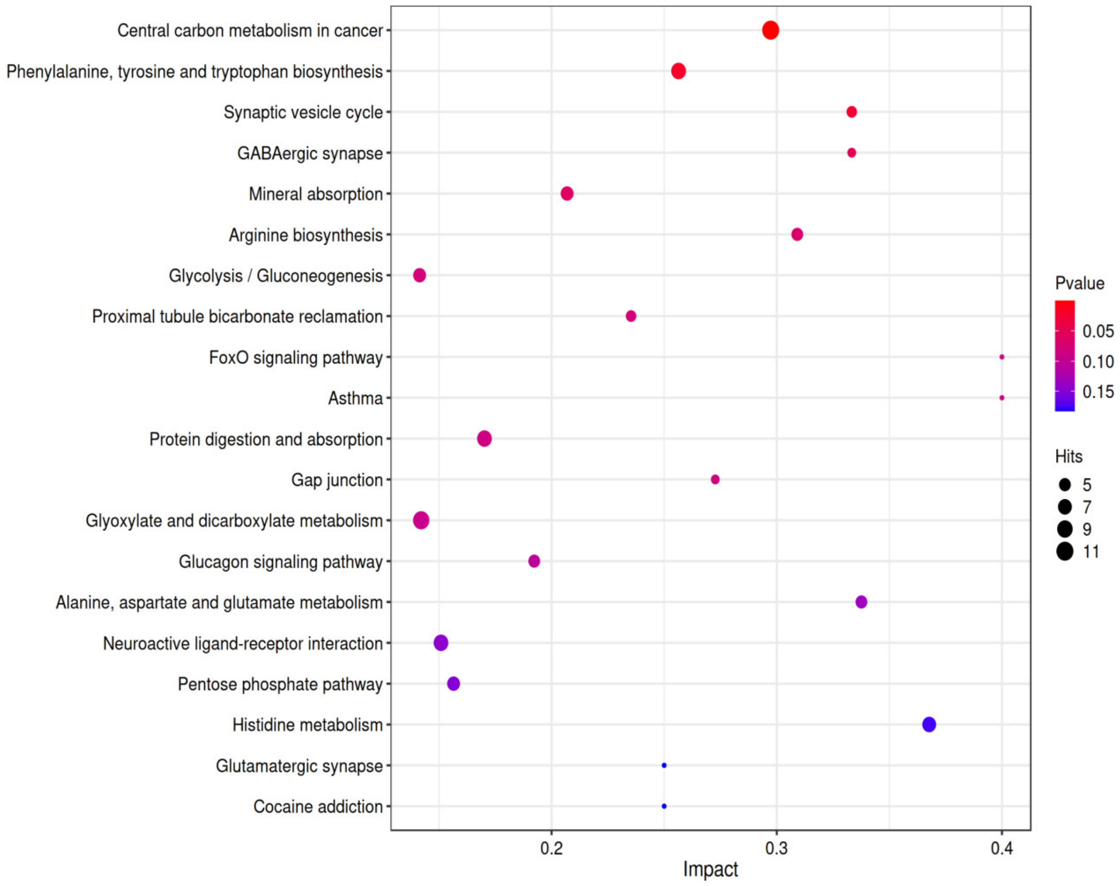

Fig.S3 KEGG enrichment analysis of differential metabolites
